# Drought Stress and Root-Associated Bacterial Communities

**DOI:** 10.3389/fpls.2017.02223

**Published:** 2018-01-09

**Authors:** Dan Naylor, Devin Coleman-Derr

**Affiliations:** ^1^Department of Plant and Microbial Biology, University of California, Berkeley, Berkeley, CA, United States; ^2^Plant Gene Expression Center, United States Department of Agriculture-Agricultural Research Service, Albany, CA, United States

**Keywords:** plant–bacteria interactions, rhizosphere, microbiome, drought stress, plant roots

## Abstract

Root-associated bacterial communities play a vital role in maintaining health of the plant host. These communities exist in complex relationships, where composition and abundance of community members is dependent on a number of factors such as local soil chemistry, plant genotype and phenotype, and perturbations in the surrounding abiotic environment. One common perturbation, drought, has been shown to have drastic effects on bacterial communities, yet little is understood about the underlying causes behind observed shifts in microbial abundance. As drought may affect root bacterial communities both directly by modulating moisture availability, as well as indirectly by altering soil chemistry and plant phenotypes, we provide a synthesis of observed trends in recent studies and discuss possible directions for future research that we hope will provide for more knowledgeable predictions about community responses to future drought events.

## Introduction

Plants are intimately intertwined with the bacterial communities found in and around their roots, which include both rhizosphere (soils in close enough proximity to the root to be influenced by root exudate release) and root endosphere (the root interior) communities (Berg et al., 2014). Plant health is closely tied to the activity of these associated microbes, and plants are known to play a role in determining the composition of their associated bacterial microbiomes (Berendsen et al., 2012). As a result of this tight interconnection, perturbations in the abiotic environment that affect either plants or their associated microbial communities can be expected to also influence the other (Wardle et al., 2004). One such wide-scale perturbation is drought, which has recently been shown to be the most influential natural disaster when it comes to agricultural productivity (Gornall et al., 2010; Lesk et al., 2016). Environmental models predict increasing frequency and intensity of drought in coming years due to global climate change (Battisti and Naylor, 2009; Lesk et al., 2016). Ongoing research conducted to understand the plant genetic mechanisms involved in tolerance to environmental stress often identifies a significant portion of missing variance attributable to the environment, the ‘environment’ in ‘genotype by environment’ interactions (Chapman et al., 1997; Xu, 2016). As one component of variance caused by the environment may be related to the plant’s microbiome, understanding exactly how drought affects root-associated bacterial communities is an essential step in developing strategies to combat drought.

Unfortunately, elucidating just how drought impacts root-associated bacterial communities is challenging due to the complexity and interconnectedness of the factors that govern the establishment of root microbiome. Plants recruit bacteria from soil communities and enrich for a host-specific root endophytic community typically of decreased diversity (Bulgarelli et al., 2012). However, this ‘starting inoculum’ of the soil microbiome will be affected by drought – both directly by selection for desiccation-tolerant taxa, as well as indirectly through altered soil chemistry and diffusion rates. Like soils, plants also undergo a set of physiological responses to drought in an effort to shield themselves from its harmful effects. These responses include alterations in root exudate profile, the primary means by which plants recruit bacteria, and in root morphology. Thus, the root microbiome under drought is determined by how drought shapes both the host plant as well as surrounding soils. To complicate matters further, each of these factors can influence the others: altered soil nutrient cycles and resulting shifts in the soil microbiome under drought will in turn have implications for plant health, as plants depend on bacterial activity to make soil nutrients bioavailable. Similarly, drought-induced changes in plant exudate profiles can alter the composition and activity of the surrounding soil microbiome, promoting further alterations to soil geochemistry that in turn alter magnitude and directionality of soil community shifts. As a result of this complexity, a truly integrated understanding of the effect of drought on the root microbiome is extremely challenging to achieve.

**FIGURE 1:**
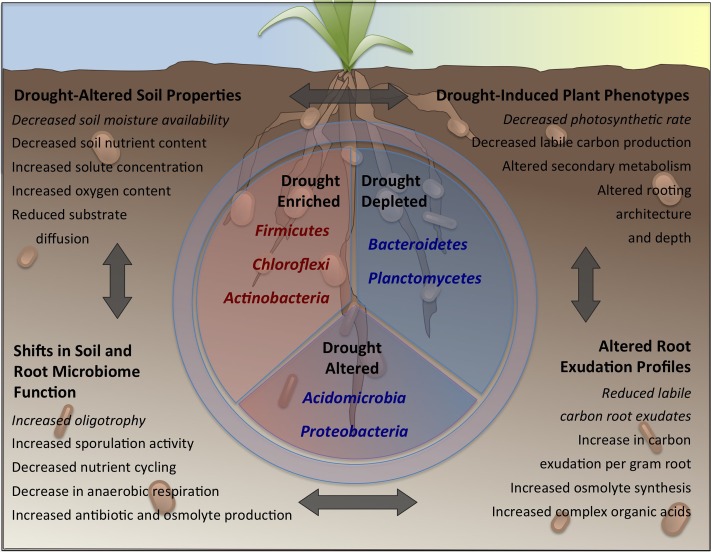
The effects of drought on soils, plants and their associated bacterial communities. Drought induces shifts in soil physicochemistry (upper left), plant phenotype (upper right), root exudation (lower right) and soil and rhizosphere microbiome function (lower left). These shifts are capable of influencing one other; for instance decreases in soil moisture availability (upper left) leads to a decrease in the rate of plant photosynthesis (upper right), which in turn leads to a reduction in the rate of labile carbon exudation to the rhizosphere (lower right) and a greater prevalence in bacteria with oligotrophic life-strategies (lower left), who are less reliant on such simple carbon sources. These shifts lead to a selection for specific phyla (center panel) within the soil, rhizosphere and root microbiome, including enrichment for many Gram-positive, oligotrophic (middle left) phyla, and concurrent depletion of many Gram-negative, copiotrophic (middle right) phyla. Members of other phyla exhibit a more balanced mixture of enrichment and depletion (middle bottom).

In this review, we address the complex interplay between soil, plant and microbe that together determine the dynamics in the root microbiome during drought (**Figure 1**). While we attempt to synthesize the available literature by grouping results into commonly observed trends in several major topical areas, we acknowledge that there are many factors with potential influence on soil and root microbiomes which we do not cover in great detail, including climatic variables, host genotype and host developmental stage. However, we propose that a first step is establishing an understanding of the effects of drought on soils and on plant physiology. The first section focuses on drought-induced compositional and functional responses of soil bacterial communities, with an emphasis on non-plant associated soils, and discusses possible reasons for the observed responses. In the second section we consider how root-associated communities shift under drought, we address the plant’s physiological responses to drought and how these exert an impact on root-associated communities, and we discuss the role of specific plant growth-promoting traits in the selection of drought-enriched taxa. We conclude with a discussion of technical challenges and limitations in current research approaches used to study the plant root microbiome under drought, and offer suggestions on directions for future research.

## Soil Bacteria Community Responses to Drought

Given that soils are the primary repository from which roots recruit their microbiomes, the drought-treated root microbiome is heavily dependent on the response of soil bacterial communities to moisture limitation. It should be noted that the term ‘soil’ in the context of microbiome studies may be used to refer to root zone soil, rhizosphere soil, or bulk soil, where the latter is assumed to be largely free of direct root influence and has higher diversity than rhizosphere soil (Lundberg et al., 2012). For consistency, research cited in this review concerning soil microbiomes was conducted on non-plant associated bulk soil (except where noted); however, it is worth noting that in some environments, the methodology of bulk soil collection occasionally necessitates removal of root tissue from soil samples, and therefore presence of root tissue may present a potential confounding factor in such analyses. Furthermore, we consider only changes associated with bacteria and refrain from addressing changes in fungal communities, as by and large the effect of drought on soil or root fungal communities is small or non-existent (Yuste et al., 2011; Bouasria et al., 2012; Barnard et al., 2013; Fuchslueger et al., 2016); thus, it should be clarified that references in this text to ‘microbes’ or the ‘microbiome’ are exclusively referring to bacterial communities. Here, we summarize the observed trends in microbial biomass, diversity, composition and activity in soil microbial communities following drought treatment, and describe potential causes of these shifts, focusing first on potentially direct causes due to a loss of soil moisture, and second on indirect causes mediated through changes in soil physicochemistry.

In general, total bacterial biomass has been observed to go down under drought (Hueso et al., 2012; Alster et al., 2013) as well as in more arid soils along a precipitation gradient (Bachar et al., 2010), as a consequence of resource limitation. That being said, in certain cases soil bacterial biomass remains stable under drought (Hartmann et al., 2017) or goes up (Fuchslueger et al., 2014), possibly due to attenuation of bacteria to repeated drought exposure (Hueso et al., 2011) and/or altered functional potential to aid in resilience (Bouskill et al., 2016b). A confounding factor may be the methodology by which bacterial biomass is determined: one method is quantification of microbial DNA (Kassem et al., 2008), whereas other studies rely on soil phospholipid fatty acid (PLFA) content (Fuchslueger et al., 2014). A definitive explanation for the observed trends in soil bacterial biomass has yet to be elucidated.

Community diversity represents another metric often applied in soil microbiome studies, where greater diversity is generally thought to be beneficial for the soils as a whole: increased species richness allows for more metabolic activities to be present, facilitating more efficient nutrient mineralization and decomposition of organic matter (Nautiyal and Dion, 2008). Overall, drought appears to have little impact on bacterial phylogenetic diversity for soil communities (Bachar et al., 2010; Acosta-Martínez et al., 2014; Armstrong et al., 2016; Tóth et al., 2017). This trend may be dependent on drought context, as in one study where plots exposed to drought for the first time were reduced by 40% in phylogenetic alpha-diversity compared to no observed change in pre-exposed plots (Bouskill et al., 2013). With respect to drought context, a confounding factor that may contribute to discrepancies described throughout the review is a lack of standardization with respect to drought treatment. Studies have imposed drought on soils through a variety of means, including exposing treatments to throughfall rain exclusion for varying time periods (Bouskill et al., 2013; Hartmann et al., 2013; Yuste et al., 2014; Tóth et al., 2017), collecting samples along a precipitation gradient (Bachar et al., 2010), or collecting soil samples from droughted and non-droughted time points (Acosta-Martínez et al., 2014).

In contrast to microbial diversity, community composition is significantly impacted by drought. The observed shifts in the soil microbiome under drought tend to involve changes in relative abundance, rather than outright abolition of drought-susceptible taxa and concomitant appearance of tolerant ones, which helps explain the lack of change in alpha-diversity. A widely observed phenomenon is an increase in the ratio of Gram-positive to Gram-negative bacteria under drought (Acosta-Martínez et al., 2014; Fuchslueger et al., 2014, 2016; Chodak et al., 2015). Specifically, in moisture-limited soils, commonly seen relative abundance shifts include decreases in largely Gram-negative phyla Proteobacteria, Verrucomicrobia, and Bacteroidetes (Barnard et al., 2013; Bouskill et al., 2013; Acosta-Martínez et al., 2014; Yuste et al., 2014), and increases in largely Gram-positive phyla Firmicutes and Actinobacteria (Bouskill et al., 2013; Chodak et al., 2015; Hartmann et al., 2017). Often these changes in relative abundance are driven by one or a few members of a phylum, as seen in Barnard et al. (2013); while relatively few groups had a large magnitude of change, most bacterial groups only had small shifts in response to drought. An experimental reduction of precipitation in German forest ecosystems provoked an increase of 300% for the family Micromonosporaceae, which was far more than its parent phylum Actinobacteria (Felsmann et al., 2015); another study found increases in Actinobacteria that were mainly attributable to members of order Actinomycetales (Bouskill et al., 2013).

It is worth noting that these taxa-specific abundance changes in soil bacteria under drought are, to an extent, context-dependent: phyla including Proteobacteria, Planctomycetes, and Acidobacteria have shown varying trends in response to water limitation. While Proteobacteria has been shown to accumulate in irrigated or non-arid soils (Bachar et al., 2010; Yuste et al., 2014; Hartmann et al., 2017), in other studies it decreases under these conditions (Bouskill et al., 2013; Acosta-Martínez et al., 2014). Another example is Acidobacteria: while this phylum has been shown to be better represented in droughted roots (Desgarennes et al., 2014) and soils (Yuste et al., 2014), it is also believed to be highly drought-sensitive (Acosta-Martínez et al., 2014) and decreases in abundance under soil dry-down (Barnard et al., 2013). Complicating matters further, Acidobacteria has been shown to have roughly equal numbers of OTUs associated with irrigated and non-irrigated soils (Hartmann et al., 2017). Such discrepancies might be explained by the relative abundance of sub-phyla in each of these studies; for example, different Acidobacteria groups display contrasting abundance shifts between control and water-excluded plots (Bouskill et al., 2013), possibly due to occupying disparate ecological niches with contrasting life-strategies (Hartmann et al., 2017) or having distinct morphologies, such as cell membrane structure, that contribute to different tolerances to desiccation.

## Potential Causes of Soil Community Trends Under Drought

A number of putative causes for the aforementioned shifts in soil community composition have been hypothesized. First, differences in substrate preference and metabolic capacities between Gram-positive and -negative bacteria may explain their distinct drought susceptibilities. Droughted environments are characteristically ‘oligotrophic’: that is, nutrient-poor but oxygen-rich. Microbes that thrive under these conditions (‘oligotrophs’) are known for being slow-growers, but can sustain growth under poor conditions. They also tend to be specialists in terms of substrate utilization (Kurm et al., 2017), rather than the more generalist copiotrophs that thrive under nutrient- and water-rich conditions such as increased litter fall after rewetting (Pascault et al., 2013; Hartmann et al., 2017). In droughted soils there is a greater abundance of bacterial genes involved in the degradation of complex plant polysaccharides and a decreased abundance targeting oligosaccharides (Bouskill et al., 2016b; Martiny et al., 2016), suggesting proliferation of oligotrophic bacteria. The oligotrophic-copiotrophic distinction overlaps with that of Gram-positive and Gram-negative bacteria, as Gram-positive bacteria are more metabolically ‘hardy’ than Gram-negative bacteria: they can utilize inorganic nitrogen to produce extracellular enzymes that degrade complex organic compounds that are relatively abundant in droughted soils (Treseder et al., 2011). For example, genera within the Gram-positive phylum Actinobacteria are capable of utilizing recalcitrant carbon sources and are highly present in arid, nutrient-poor soils (Connon et al., 2007; Yuste et al., 2014; Mohammadipanah and Wink, 2016; Hartmann et al., 2017). By contrast, Gram-negative bacteria contain characteristics of copiotrophs, as they prefer labile carbon compounds and organic nitrogen (Treseder et al., 2011), particularly in the form of plant root exudates (Balasooriya et al., 2014); and indeed it has been observed that Gram-negative bacteria incorporated almost ten times as much plant-derived carbon as Gram-positive bacteria under well-watered conditions (Fuchslueger et al., 2014). Under drought, labile organic carbon is increasingly scarce within soils (Thaysen et al., 2017), and in turn the rate of transfer of plant-derived carbon to microbes has been observed to go down (Ruehr et al., 2009), possibly as microbial communities switch to degrading more recalcitrant carbon sources within plant organic matter (Bradford et al., 2008). Karst et al. (2016) posited that plants close protein channels to prevent sugar transport to the rhizosphere as part of osmotic adjustment under drought. However, while lifestyle differences might partially explain the observed enrichment patterns, certain phyla that are predominantly Gram-negative or -positive are not universally copiotrophic or oligotrophic (Fierer et al., 2007), suggesting other factors likely also contribute.

A second putative and more direct cause of the altered soil community composition under drought is related to differences in tolerance to loss of soil moisture. The various physiological mechanisms that allow bacteria to tolerate drought, including sporulation and thick cell walls, are not evenly distributed between bacterial groups. Many genera within primarily Gram-positive phyla are known sporulators, while Gram-negative phyla largely lost the capacity to sporulate during the course of evolution (Tocheva et al., 2016). Sporulation allows bacteria to enter dormancy under periods of environmental stress and has been posited as a contributing factor for observed abundance trends (Hayden et al., 2012; Marasco et al., 2012; Acosta-Martínez et al., 2014). Additionally, Gram-positive bacteria are characterized by a thicker peptidoglycan cell wall layer than that of Gram-negative bacteria, which may render them more resistant to drought (Schimel et al., 2007). The correlation between cell wall thickness and Gram-staining is not universal, however, there are numerous exceptions of thin-walled taxa within primarily Gram-positive phyla and vice versa, and for this reason it may be advisable to consider the role of physiology in drought resistance in terms of thick-walled (monoderm) vs. thin-walled (diderm) taxa, in particular as the largely monoderm phyla have been demonstrated to have far drier optimal environmental niches than largely diderm phyla (Lennon et al., 2012).

A third hypothetical cause of the drought-induced shifts in community composition is related to levels of general and specific bacterial activities. Overall bacterial activity is positively correlated with moisture availability, to the point where a massive increase in activity and CO_2_ efflux is observed after rewetting of dry soils (Blazewicz et al., 2014; Armstrong et al., 2016), a phenomenon known as the ‘Birch effect.’ In wetter soils, gaseous diffusion into and out of soil is reduced, creating a more anaerobic environment (Liptzin et al., 2011) and thus higher bacterial gene abundances for genes involved in anaerobic fermentation (Schwartz et al., 2007), O_2_ limitation and other anaerobic processes such as denitrification are observed (Bouskill et al., 2016b). By contrast, under drought soil bacterial activity tends to decrease as microbes die or enter dormancy (Jensen et al., 2003; Alster et al., 2013), although elevated gene abundance for complex carbon degradation has been observed (Bouskill et al., 2016b). As a result, bacterial groups containing activities favored under a given moisture level may be enriched under such conditions.

In addition to the above, a range of other activities may play a role in community trends. First, drought may induce existing microbes to produce a variety of compounds that affect community stability. For instance, drought-treated soils contain more antibiotics, which are hypothesized to be produced by drought-tolerant bacteria as a physiological response to outcompete other bacteria for limited resources, or possibly as signals to induce drought-response pathways like biofilm formation (Bouskill et al., 2016a). Additionally, certain bacteria synthesize compounds during drought that influence rhizosphere soil aggregate stability (Kohler et al., 2009) and hydrophobicity (Elbl et al., 2014). Finally, differences in the ability to produce and accumulate osmolytes, which maintain cellular turgor and protect macromolecular structures (Welsh, 2000), may play a role. These compounds include amino acids, such as proline, glutamine, and glycine betaine, and carbohydrates, such as trehalose and ectoine (Bouskill et al., 2016b). It has been shown that Gram-negative bacteria produce osmolytes purely as a drought-inducible response, whereas Gram-positive bacteria tend to produce osmolytes, at least partially, on a constitutive basis (Schimel et al., 2007). Synthesis of osmolytes is metabolically demanding; it is estimated that 3–6% of net primary production in a grassland ecosystem can be consumed by microbes within a single drought event (Schimel et al., 2007). As a result of this increased demand for carbon, enzyme activities related to depolymerization of plant macromolecules are elevated in drought-treated soils than control (Bouskill et al., 2016b). The observed enrichment for Gram-positive bacteria, especially lineages such as Actinobacteria that contain genes for complex carbon degradation, is in line with these observations (Mohammadipanah and Wink, 2016). Future study of microbial functional capacity will help identify specific activities that are enriched or depleted under drought and how such shifts contribute to observed community abundance trends.

Indirect causes mediated by changes in the soil physicochemical properties are also known to play a role in shaping microbial communities. For instance, low soil moisture reduces soil pore connectivity, raises solute concentrations in the remaining water, and limits substrate diffusion (Schimel et al., 2007; Liptzin et al., 2011). Water-limited soils may be decreased in overall ion content including calcium carbonate, sodium, potassium (Bachar et al., 2010), phosphorus, and other redox-sensitive compounds (Al, Fe, Mo) (Bouskill et al., 2016b). These changes in soil chemistry will exert an influence on the microbiome – in experiments analyzing the influence of various factors on soil bacteria, chemical properties including pH and ion content were significant in determining community composition (Lauber et al., 2009; Gunnigle et al., 2017; Hartmann et al., 2017), often on a taxa-specific basis. For example, when looking at Mediterranean forest sites with different soil moisture levels (in which ion content decreased along the precipitation gradient), abundance of phylum Acidobacteria correlated positively with soil ammonium and phosphorus and negatively with nitrate and magnesium, whereas phylum Chloroflexi displayed the opposite trend (Bachar et al., 2010). It is worth noting that the influence of soil chemistry is an important albeit sometimes overlooked factor, and that identical drought treatments placed upon chemically distinct soils will induce different responses in their respective bacterial communities – factors including pH, total nitrogen, and organic carbon content have been shown to influence how different bacterial phyla respond to drought and rewetting (Chodak et al., 2015).

One challenge in establishing an unconditional link between drought-induced changes in soil chemistry and bacterial communities is that bacteria themselves may play a role in soil chemical cycles (Coleman et al., 1983). Under water stress, rates of microbial enzymes responsible for biogeochemical nutrient cycling and decomposition go down (Stark and Firestone, 1995; Hueso et al., 2012), likely due to a combination of limited substrate supply due to decreased diffusion (German et al., 2011), intracellular accumulation of ions and osmolytes to levels inhibitory toward enzymes, as well as lower enzymatic hydration and altered conformation (Csonka, 1989). For example, an experimental 10% reduction in soil moisture significantly decreased the rates of nutrient cycling enzymes, an effect that was enhanced by increasing the reduction to 21% (Sardans and Peñuelas, 2005). In another study, when fertilizer was applied to soil plots that were subsequently exposed to drought, there were vast increases in soil ammonium and nitrate, which were hypothesized as being a result of reduced nitrification activity in soil microbes (Hartmann et al., 2013). Additional studies have confirmed a decline in nitrification (Stark and Firestone, 1995; Ford et al., 2007), phosphorus solubilization (Sardans and Peñuelas, 2004), and carbon cycling (van der Molen et al., 2011; Hueso et al., 2012). Likely due to reduced nutrient cycling, droughted soils have been shown to contain lower levels of soil nitrate (Fuchslueger et al., 2014), soil-available forms of phosphorus (Sardans and Peñuelas, 2004), as well as increases in complex organic carbon (Sardans et al., 2008; Hueso et al., 2012). However, it is important to note that in some studies a less pronounced correlation has been found between soil moisture and nutrient cycling activities, perhaps as a consequence of additional confounding factors, such as temperature and season (Kramer and Green, 2000; Sardans et al., 2008), that were not controlled or measured in these studies.

It should be noted that several important and often overlooked factors might contribute to discrepancies observed across and within soil microbiome studies. First, studies are often conducted at different times or seasons: when studying the soil microbial community drought response over the course of a year, a number of enrichment patterns were more pronounced during summer than winter and spring seasons (Yuste et al., 2014). Even over the course of a single day, differences in Actinobacteria and Proteobacteria can be seen, which is at least in part due to fluctuating microenvironmental parameters including soil humidity, which was found to differ by 34% over the course of a 24-h cycle (Gunnigle et al., 2017). In the case of field studies, differences in the frequency of drought between fields may also represent a confounding factor, as repeated exposure to drought can ‘attenuate’ soils to future drought events (Cruz-Martínez et al., 2009). Soil respiratory responses, as well as soil physical and chemical responses, are less pronounced in previously drought-exposed soils compared with soils not exposed to drought beforehand (Göransson et al., 2013; Bouskill et al., 2016a). With respect to community responses, decreases in phylogenetic diversity (relative to control plots) were seen only in experimental plots exposed to drought for the first time, and not those with drought history – perhaps due to sensitive taxa evolving drought resistance (Bouskill et al., 2013), or accumulation of communities that remain robust against changing soil water dynamics (Cruz-Martínez et al., 2009). The precise mechanism by which bacterial resistance improves is unclear. However, soils with drought history have decreased presence of plant-derived carbon in microbial biomarkers, suggesting that attenuation to drought in the soil microbes includes a decreasing dependence on plant carbon sources (Fuchslueger et al., 2016) and selection for bacteria with an oligotrophic lifestyle, as discussed earlier. Thus, while broad and common trends in bacterial abundance can be observed within soils under drought, many additional environmental factors may reduce or reverse these patterns. In the future, additional research to identify missing environmental parameters that play roles in shaping soil microbiomes under drought is needed.

## Plant Responses to Drought

While changes in the surrounding soil chemistry and soil microbiome composition and activity can be expected to alter the available pool of bacteria from which plants recruit endophytic communities, drought-induced changes in plant physiology and biochemistry are perhaps even more influential on root microbiome dynamics. Such changes include alterations in root morphology, overall carbon efflux into the soils, and the root exudate profile. In looking at the effect of soil moisture on the root microbiome, experiments have been conducted in the context of experimental drought, but also in the context of season (i.e., dry vs. rainy), the latter being more common (Torres-Cortés et al., 2012; Nessner Kavamura et al., 2013; Desgarennes et al., 2014; Coleman-Derr et al., 2016; Fonseca-García et al., 2016; Taketani et al., 2017). For the purposes of this review, both contexts will be considered, as the available literature on experimental drought’s effects on the root microbiome is limited. Similarly, in the following sections we include discussion of both rhizosphere and root endosphere microbial communities, differentiating between the two where applicable. While each compartment may exhibit some distinct trends from the other, they are both subject to significant influence by the root and fall under the heading of ‘root-associated’ communities; furthermore, research conducted on the effect of drought on either compartment alone is limited, and considering them in conjunction can help us to establish general trends otherwise obscured by current knowledge gaps. Here, we first summarize the observed trends in microbial composition and activity in root-associated microbiomes following exposure to drought, and then we explore plant responses to drought that might influence the root microbiome and discuss the potential benefit that plants receive from recruitment of specific bacterial lineages.

Seasonality, specifically the ‘dry’ vs. ‘rainy’ season division, has been demonstrated as a statistically significant factor in determining root-associated microbial community composition (Taketani et al., 2017) [albeit in some cases exerting a relatively small influence (Desgarennes et al., 2014)]. In some cases, the relative strength of this effect depends on host species or compartment. In studies on wild and cultivated *Agave* species, season was the greatest contributing factor to variance in the root endosphere microbiome, whereas the rhizosphere and leaf phyllosphere were primarily influenced by host species (Desgarennes et al., 2014; Coleman-Derr et al., 2016). Heightened influence by season within the root endosphere was also seen in cacti (Fonseca-García et al., 2016) and the tree species *Populus deltoides* (Shakya et al., 2013), which may reflect the increased plant–microbe intimacy inside roots, and that the plant’s responses to drought will be most likely to influence these communities as compared to external ones. Another study found season was a significant factor on *Agave* bacterial communities, but only for cultivated species (Coleman-Derr et al., 2016), suggesting wild species’ bacterial communities are more resistant to changes in season and therefore water availability.

An important caveat to note is while some of these papers noted seasonal differences in precipitation (Desgarennes et al., 2014; Coleman-Derr et al., 2016; Fonseca-García et al., 2016) or soil moisture around the base of the plant (Shakya et al., 2013), ‘drought’ and ‘season’ are not synonymous, and such experiments can provide only indirect connections between community composition and water content, as there may be confounding factors that differ by season. As a result, studies looking at experimental manipulations in soil moisture within a single time frame are preferable. Experimental designs in such studies have included measuring soil water content and including that as an explanatory factor (Marschner et al., 2005; Nuccio et al., 2016), or experimentally manipulating irrigation to artificially impose drought (Cherif et al., 2015; Naylor et al., 2017; Santos-Medellín et al., 2017) [however, it should be mentioned that in the latter case, there exists a lack of consistency to the definition of drought, ranging from intermittent watering (Cherif et al., 2015; Santos-Medellín et al., 2017) to complete cessation of irrigation during the drought treatment (Naylor et al., 2017)]. Variations in soil moisture content within a season have been shown to be a significant factor in determining bacterial community composition in root and/or rhizosphere communities, as shown in the wildflower genus *Banksia* (Marschner et al., 2005) and date palms (Cherif et al., 2015). In fact, a study looking at grassland rhizosphere communities found of all soil properties, it was gravimetric water content at sampling that had the greatest effect on the rhizosphere microbiome community (Nuccio et al., 2016). Analysis of cereal grasses found that drought regime was the second-greatest contributing factor to beta-diversity after plant compartment (Naylor et al., 2017). A study of four distinct rice genotypes in three soil types again found drought to be a significant factor, and that directionality in drought responses in rhizosphere and root endosphere bacterial communities were largely conserved between soil types (Santos-Medellín et al., 2017), implicating the plant host as an important player in such responses.

Whether as a consequence of recruiting from drought-affected soil communities, or due to endophytic communities experiencing similar responses to those in the exterior environment, it has been shown that changes in relative abundance in the root microbiome are largely similar to those seen in soil. Like in soil, during the dry season root-associated communities show elevated abundance for Actinobacteria, Acidobacteria, and Bacillus, whereas in the rainy season Proteobacteria and Bacteroidetes are enriched; these trends have been demonstrated for rhizosphere communities in the tree species *P. deltoides* (Shakya et al., 2013) and *Mimosa tenuiflora* (Taketani et al., 2017), and in the cactus *Cereus jamacaru* (Nessner Kavamura et al., 2013), suggesting the inherent qualities that cause enrichment by a given water strategy in the soil are not circumvented by the change in local environment. However, in two recent direct manipulation experiments, higher levels of drought enrichment were observed for members of the Actinobacteria and Firmicutes (Naylor et al., 2017; Santos-Medellín et al., 2017) within root endosphere and rhizosphere communities as compared to the surrounding soil. These studies suggest that while similar taxonomic trends may be observable between soil and root communities exposed to drought, the degree of enrichment and in some cases the specific taxa may differ.

Interestingly, identification of a ‘core microbiome’ of drought-enriched taxa in root endospheres in several different studies (Desgarennes et al., 2014; Coleman-Derr et al., 2016; Naylor et al., 2017) found that these cores contained numerous members of classes Alpha-, Beta-, and Gamma-proteobacteria, in addition to more commonly drought-associated Gram-positive lineages. These trends might reflect laxity in the parameters used to obtain the core microbiome, as well as disparity between sites [(Coleman-Derr et al., 2016) used different field sites to grow their three *Agave* species]. Alternatively, there may also be innate plant growth-promoting (PGP) properties in the enriched taxa within Proteobacteria, such as nutrient solubilization or presence of ACC deaminase, which plants select for under drought. Indeed, as broad trends of enrichment are not necessarily universally true for all members of a phylum, it is feasible that specific outlying lineages may be actively recruited by the root based on the presence of specific PGP traits despite harboring a degree of drought sensitivity.

In addition to shifts in community composition, drought will induce shifts in the functional profile of the rhizosphere, as plants are implicated in recruiting beneficial bacteria in response to drought (Marasco et al., 2012). Rhizosphere bacterial enzymatic activity is generally higher than that of bulk soil (Marschner et al., 2005), as is functional diversity (Li et al., 2014), suggesting part of bacterial enrichment around plant roots is for their functional capacity. da Costa et al. (2014) hypothesized that under nutrient-rich conditions plants will favor recruitment of phytohormone-producing microbes, while in nutrient-poor conditions plants will tend to favor interactions with nutrient solubilizers, as soil mineralization and plant uptake of nutrients are impaired under drought (He and Dijkstra, 2014). Diazotrophic bacteria, which solubilize nitrogen and have demonstrated capacity to enhance plant growth (Knoth et al., 2014), are associated with *Agave* roots under drought (Desgarennes et al., 2014). Similarly, comparative analysis of corn rhizospheres under drought found higher protease, catalase, alkaline phosphatase, and invertase activities at most growth stages in drought-tolerant cultivars compared with drought-susceptible cultivars, a discrepancy they posited was attributable to differences in root exudates and microbial community composition (Song et al., 2012).

An important question is whether the observed shifts in root microbiome composition and activity are conserved across the plant kingdom. In a study comparing the effect of various experimental factors on the rhizosphere bacterial communities in four grass species, while both host species and watering regime affected beta-diversity, the drought effect was particularly pronounced for the more drought-susceptible species (Bouasria et al., 2012). Similarly, when comparing local soil communities in the presence of three common pasture plants, bacterial resistance and resilience to drying was distinct between the tested plant species (Orwin and Wardle, 2005). By contrast, when comparing rice varieties, drought responses were largely comparable between genotype (Santos-Medellín et al., 2017), suggesting that more genetically distinct plant species should be included to allow for observations of more distinct drought trends by host species, as root and rhizosphere communities are more similar for closely related lineages (Peiffer et al., 2013; Bouffaud et al., 2014).

The host species effect under drought is perhaps best studied in Naylor et al. (2017), in which 18 different grass accessions and outgroup tomato were grown in a common field and exposed to drought. Host species was confirmed to exert a significant influence on beta-diversity in both control and droughted rhizosphere and root endosphere communities. While the specific abundance trends of bacterial taxa under drought by plant species were not extensively studied, some broad trends were noticeable – for example, the three sorghum lines studied (two accessions of *Sorghum bicolor*, as well as *Sorghum laxiflorum*) had more unique droughted root core microbiome, sharing much fewer of their core OTUs with other species’ cores. Perhaps most interestingly, C4 grasses had more pronounced average drought enrichment (3.4-fold) for class Actinobacteria than C3 grasses (2.4-fold). Given that Actinobacteria are implicated in promoting plant growth under stress (Yandigeri et al., 2012; Anwar et al., 2016), heightened enrichment in C4 grasses (whose range includes more arid and semi-arid regions than C3 grasses) may be part of evolved tolerance to drought-prone habitats. Thus, while we can draw broad conclusions about common trends in the root microbiome under drought, it should be stressed that they should be taken with the caveat that factors including host genotype, as well as duration and type of drought treatment, can all influence observed outcomes.

## Causes for Bacterial Community Trends in Drought-Stressed Roots

Plants have evolved complex morphological and metabolic responses to drought stress, many of which have been hypothesized or demonstrated to play a role in shaping root associated microbial communities. The full contingent of metabolic mechanisms plants use to deal with low water availability have previously been extensively reviewed (Fang and Xiong, 2015), and many of these responses overlap with those of bacteria. For instance, both plants and bacteria alter metabolism in accordance with available carbon pools, synthesize osmolytes to reduce osmotic stress, and activate stress pathways, such as antioxidant defense. One plant process that is particularly affected is photosynthesis, where reduced stomatal conductance and lowered photosynthetic capacity have been observed under drought (Albert et al., 2011), as the result of decreased chlorophyll content, fluorescence, and quantum yield (Ghotbi-Ravandi et al., 2014; Khan et al., 2016). Upregulation of chlorophyll synthesis is a common drought tolerance strategy in plants (Fang and Xiong, 2015) – drought resistance in certain cultivars of barley and sorghum has been attributed to their ability to ameliorate photosynthetic inhibition through retaining chlorophyll content and CO_2_ assimilation (Ghotbi-Ravandi et al., 2014; Ogbaga et al., 2014).

As a consequence of these shifts in photosynthesis, changes in the plant metabolomic profile are common under drought – one study identified 163 metabolites that significantly change in abundance during water stress in roots (Tripathi et al., 2016). Compounds with upregulated synthesis include sugars, polyols, amino acids, alkaloids, and ions, which help with maintaining photosynthesis, cell osmolarity, as well as delaying leaf senescence and enhancing root growth (Fang and Xiong, 2015). Additionally, increased synthesis in cell wall polymers helps to maintain cell turgor and strengthen the cell wall (Gall et al., 2015); to that end, syntheses of xyloglucan, expansin, pectins, lignin, and suberin are shown to be upregulated under drought (Jones and McQueen-Mason, 2004; Cho et al., 2006; Moura et al., 2010; Peaucelle et al., 2012).

The rate of translocation of newly assimilated carbon from shoots to roots has been hypothesized to go down under drought (Hasibeder et al., 2015), as plants close protein channels and shift carbon toward production of osmolytes and storage compounds. Concurrently, bacteria groups largely reliant on plant carbon will die or decrease in abundance (Fontaine et al., 2003), and remaining bacterial activity is restricted to isolated areas of moisture such as soil pores, hindering their ability to interact with plants (Schimel et al., 2007). Drought alters plant carbon output into the soil (Ruehr et al., 2009; Albert et al., 2011) – multiple studies where radiolabeled carbon was supplied to plants under drought observed reduced uptake of tracers in soil bacteria compared to control conditions (Ruehr et al., 2009; Fuchslueger et al., 2014), an effect exacerbated in soil plots with previous drought history (Fuchslueger et al., 2016). However, the methodologies used in these studies may have been fundamentally flawed: observations of decreased carbon flux from plants to soils under drought may be partially attributable to failure to account for changes in root production. When corrected for, it has been found carbon flux was not affected by drought (Canarini and Dijkstra, 2015). One review (Preece and Peñuelas, 2016) systematically compared studies, finding that when a decrease in plant biomass is accounted for, moderate drought tends to increase carbon flux into the soil *per gram of plant* (although severe drought can halt or even reverse this trend, suggesting that there is a ‘threshold’ of drought). Furthermore, the proportion of carbon allocated to roots vs. shoots increases (Palta and Gregory, 1997), especially with increasing drought stress dose (Zang et al., 2014), suggesting that rather than a breakdown of the relationship between plants and bacteria, instead it is enhanced under drought. Increases in carbon efflux to soils could implicate the plant in altering its root communities in response to drought, considering the importance of root exudates in microbial recruitment.

Carbon efflux from the plant to the soil may take several forms (including release of dead cell contents, VOC emission, and transfer of carbon to microbial symbionts), but of these forms, root exudates are most directly implicated in recruitment of the root microbiome. Root exudates are carbon-containing compounds [ions, sugars, amino acids, enzymes, organic acids, and mucilage (Preece and Peñuelas, 2016)] released from roots either indirectly (i.e., from senescing roots and/or lysis of root cells) or directly through a process known as ‘rhizodeposition.’ Root exudates are considered to ‘prime’ the soil environment around the roots – that is, they will attract beneficial bacteria to the rhizosphere, thereby increasing respiration rates and percentage of bacterial biomass in rhizosphere compared to bulk soil (Nannipieri et al., 2007). In doing so, rates of soil mineralization and soil organic matter decomposition go up (Fontaine et al., 2003; Fuchslueger et al., 2014), which benefits both plants and bacteria.

While between 64 and 86% of characteristic plant rhizodeposits are capable of being respired by microorganisms (Hutsch et al., 2002), recruitment patterns of root-associated bacterial communities are highly dependent on the exact root exudate profile (Badri et al., 2013) – for example, various *Arabidopsis* accessions have distinct exudate profiles, and both root and rhizosphere bacterial communities were in turn found to be distinct between these accessions (Micallef et al., 2009). Due to the complexity of the microbiome, it is difficult to elucidate a connection between a given exudate and which microbe(s) it recruits. Instead, broad changes in community abundance and diversity have been reported. Organic acids, and to a lesser extent sugars, increase overall bacterial richness in the rhizosphere community, and varying effects were found between the individual organic acids (Shi et al., 2011). Results from Badri et al. (2013) imply that sugar, sugar alcohols, and amino acids are broad-range attractants while phenolic compounds recruit bacterial taxa in a more specific manner. While it is difficult to correlate exudate profile changes with particular community responses, it has been shown that exudate profiles differ under drought, which will have significant implications for root communities.

Firstly, as previously mentioned, cumulative organic carbon exudation per gram dry plant increases up to 71% under drought (Henry et al., 2007), though much like growth, this response is attenuated by increasing severity of the stress (Reid, 1974). More specifically, in Calvo et al. (2016) barley plants under reduced water supply exhibited greater proline, potassium, and phytohormone concentrations in root exudates, which have roles in enhancing root growth, osmoprotection, and stress signaling, if not necessarily bacterial recruitment. Elevated presence of organic acids (fumaric acid, succinic acid, oxalic acid, malonic acid, and malic acid) (Henry et al., 2007), water-soluble carbon, mucilage, sterols, and polar lipids (Whipps and Lynch, 1983; Svenningsson et al., 1990) have been seen around drought-stressed roots. The exudate response can be distinct between plant species (Canarini et al., 2016) or even cultivars – rhizodeposition increases under drought for monocots, while it tends to decrease for dicots; similarly, cultivated species have a less pronounced change than wild species (Preece and Peñuelas, 2016). In one study comparing drought-tolerant and -susceptible corn hybrid cultivars (Song et al., 2012), the former exuded greater quantities of organic acids (in this case, lactic, acetic, citric, and maleic acids) under drought.

Taken together, the studies above implicate organic acids as part of the root exudate profile response to drought. Organic acids promote drought tolerance for the plant independently of bacteria, through solubilization of nutrients such as iron, manganese, and phosphorus, among others (Delhaize et al., 1993; Ström et al., 2002). But, as discussed above, organic acids are implicated in recruiting bacteria in distinct ways (Shi et al., 2011) – for example, a positive correlation was seen for exudation of salicylic acid and GABA with Actinobacteria and other ACC deaminase-producing bacteria (Badri et al., 2013). Apart from organic acid release, efflux of hydrogen peroxide has been implicated as a means of plant drought tolerance to protect against ROS damage (Huang et al., 2017) and maintain apical root growth (Voothuluru and Sharp, 2012), which may explain increased prevalence of Actinobacterial genera such as *Streptomyces* around droughted plant roots and rhizospheres (Naylor et al., 2017), as many *Streptomyces* lineages are able to effectively reduce ROS damage in plants (Lee et al., 2005; Leiros et al., 2014). More research will be needed to make explicit connections for microbial recruitment patterns by plant root exudates under drought.

These shifts in plant metabolism and exudation are mirrored by changes in plant morphological responses, which include leaf rolling, stomatal closure, decreased leaf area, increased synthesis of water-storing tissues such as tubers, and wax accumulation on the leaf surface. However, given that the phenotypic changes that will have the greatest impact on the root microbiome will naturally be in the roots, here we focus on those changes. Root morphology is highly associated with drought resistance, as longer and more extensive root systems allow plants more opportunities to take up water and nutrients. Drought-tolerant plants will tend to have a greater rooting depth, density, root volume and weight (Fang and Xiong, 2015). Grasses such as sorghum that evolved in more arid regions display a more vertical root morphology and deeper rooting depth than grasses such as maize that evolved in more temperate regions (Singh et al., 2010). Under drought, a common plant response is to enhance root growth to maintain water uptake, even as shoot growth is hindered (Spollen and Sharp, 1991), although under severe drought, root growth is severely abated (Zang et al., 2014). Plants may dynamically modify root architecture to account for limited water availability. Aspects of soybean root architecture (depth, branching density, root angle, ratio of root to shoot biomass) are all affected by drought (Fenta et al., 2014). Root modification may even occur in a species-specific manner (Hartung and Turner, 1997; Bouasria et al., 2012; Smith and De Smet, 2012). Changing root morphology – in particular rooting depth – may alter the composition of the bacterial communities, as soils at different depths have their own characteristic bacterial community patterns (Delmont et al., 2012; Zhang et al., 2017) and furthermore rhizospheres from root sections taken at different depths have distinct microbiota (Kawasaki et al., 2016).

## The Role of Bacteria in Plant Growth Promotion Under Drought

Plant recruitment of a drought-specific microbiome could be an evolved trait, where generations of repeated drought events have led to evolution of stable and beneficial plant-microbe interactions that improve the reproductive fitness of both host and microbe. In one study, *Brassica rapa* plants that had been exposed to generations of drought were better able than control plants to increase bacterial abundance and diversity around roots under dry contemporary environments (TerHorst et al., 2014). Alternatively, a drought-tolerant community may be achieved through soil attenuation, in which bacterial communities in soils exposed to drought have developed resistance, and thus the plant will have no choice but to recruit a beneficial microbiome. This was demonstrated in Lau and Lennon (2012), where plant fitness under drought was highest when grown in previously droughted soils, while plant fitness under well-watered conditions was highest in soils where water was historically abundant. Even simply having a sympatric soil (i.e., a soil in which a given plant has been repeatedly grown in) can improve a plant’s performance (in this case, biomass and drought-responsive gene expression) under drought compared with the same conditions in non-sympatric soils (Zolla et al., 2013), suggesting that even when not under stress, plants will recruit beneficial bacteria that remain in the soil and can enhance drought tolerance for other members of their species.

By definition, a plant’s relative health and fitness increases following the recruitment of microbes with PGP activities, and engineering stable interactions between plants and a desired microbiome represents an attractive target for crop improvement through stress tolerance (Quiza et al., 2015). An exploration of previously identified plant growth-promoting microbes from drought treated plants may offer clues as to which microbial traits are likely beneficial to and potentially selected for by plants. Indeed, roots and soils are frequently found to harbor bacteria with PGP abilities (Grönemeyer et al., 2012), especially in chronically drought stressed regions (Mayak et al., 2004; Timmusk et al., 2014). For example, a survey of barley rhizospheres and bulk soil found isolates from a sunny, stressed site in Israel had a greater variety of PGP abilities than the non-stressed site’s isolates. Furthermore, enzymatic activities were much higher for rhizosphere-associated isolates compared with those from bulk soil (Timmusk et al., 2011). Interestingly, in another study looking at pepper plants, activities that affected the plant most directly, such as phytohormone synthesis, were primarily in root endophytes, whereas nutrient solubilizers were better represented in rhizosphere and bulk soil (Marasco et al., 2012).

Screening of putative plant growth-promoting bacteria (PGPB) *in vivo* on droughted plants is a frequent strategy used to confirm growth promotion, and has been done in a variety of plant species (Mayak et al., 2004; Marasco et al., 2012; Yandigeri et al., 2012; Timmusk et al., 2014; Wang et al., 2014; Cherif et al., 2015). PGPB may enhance drought tolerance in plants other than those they were originally isolated from (Marasco et al., 2013; Wang et al., 2014), and in some cases enhance plant growth only under drought conditions (Wang et al., 2014; Rolli et al., 2015). Creating consortia of bacteria may have greater and synergistic effects at alleviating drought than applications of individual genera (Knoth et al., 2014; Timm et al., 2016). A consortium of 10 endophytic strains applied to hybrid poplar enhanced plant survival under water limitation through multiple distinct drought-response pathways (Khan et al., 2016). These results serve to highlight that drought may induce the plant to accumulate bacteria with specific tolerance activities, and this accumulation occurs on a community level rather than enriching for specific genera.

A variety of PGP abilities are implicated in conferring drought tolerance, of which perhaps the most studied is the enzyme 1-aminocyclopropane-1-carboxylate deaminase (ACCd). Through ACCd activity, the plant hormone ethylene remains below inhibitory levels, maintaining normal root growth and delaying senescence under drought (Glick, 2004). PGPB are also known for synthesizing other phytohormones, including the auxin analog indole-3-acetic acid (IAA), which can enhance shoot and root growth among other plant developmental processes (Glick, 1995). During drought, PGPB may be involved in nutrient cycling, including diazotrophy, phosphorus solubilization, and siderophore synthesis (Kim et al., 2012); they have also been shown to enhance photosynthesis, increase fine root production and greater overall root surface area, and decrease stress volatile emission, all of which have demonstrated improvement of plant performance (Casanovas et al., 2002; Timmusk et al., 2014; Gagné-Bourque et al., 2016). They have even been implicated in accelerating flowering (Lau and Lennon, 2012; Gagne-Bourque et al., 2015), including earlier seed set times and senescence, as early flowering is a strategy plants can evolve as a means of drought escape (Franks et al., 2007).

While it is difficult to elucidate the explicit mechanism by which PGPB act to enhance drought tolerance, in certain cases it has been demonstrated. For example, PGPB in Sandhya et al. (2009) produced extracellular matrices to maintain a hydrated root environment, increasing root-adhering soil and stability. Similar results were seen in Timmusk et al. (2015), in which a mutant of *Paenibacillus polymyxa* lacking an Sfp-type 4′-phosphopantetheinyl transferase had heightened biofilm production, which upon inoculation on drought-stressed wheat plants was shown to enhance plant survival and biomass production two and threefold, respectively. In the presence of droughted *Arabidopsis* roots, *Bacillus megaterium* BOFC15 secretes the polyamine spermidine, which scavenges ROS, upregulates ABA biosynthesis and response genes, and by extension augments photosynthesis and root system architecture (Zhou et al., 2016). Similarly, *Pseudomonas chloroaphis* O6 will synthesize 2*R*-3*R*-butanediol, which is involved in the SA signaling pathway and elicits stomatal closure in *Arabidopsis* (Cho et al., 2008).

Altering plant gene expression is a common mechanism PGPB can use to confer drought tolerance in the plants. Often this has been demonstrated in a non-specific manner, such as upregulation of marker drought-response genes such as *DREB1B-like* or *ERD15* (Kariola et al., 2006; Gagne-Bourque et al., 2015), which have numerous downstream targets. However, it should be noted that while in some cases specific drought stress genes are expressed, there exists significant crosstalk among signaling pathways associated with abiotic and biotic stress responses, which hints at mechanisms that integrate global plant stress responses. Timmusk and Wagner (1999) suggested such a link in the very first report on rhizobacterial plant drought stress tolerance enhancement. The phenomenon is now repeatedly confirmed by numerous authors (Gassmann et al., 2016), and calls for greater collaboration among plant biologists studying different stresses, in order to address the complexity of plant stress responses under natural conditions.

## Current Limitations and Future Outlook

In this review, we have addressed the interdependent factors that determine drought response in root-associated bacterial communities. While there are promising findings that have come out of the extensive research conducted thus far, numerous limitations in experimental methodologies preclude drawing more concrete conclusions about the observed trends. Here, we present a brief overview of three of these issues and present suggestions for future research to address them.

Firstly, conclusions drawn about responses in the root microbiome to an external drought stress may be spurious when conducted in a traditional experimental framework – that is, when plants under control and drought conditions differ only by a single factor, the amount of water supplied. However, multiple environmental variables may accompany drought, including increases in temperature and soil salinity (Bartels and Sunkar, 2005; Liu et al., 2014), all of which present unique stresses and elicit different responses in plants (Meena et al., 2017). Thus, experiments incorporating multiple variables will be more valuable in determining microbiome responses in an agronomically valuable, real-world context, than experiments conducted manipulating one factor at a time. Furthermore, mathematical representations of agro-ecosystems can assist with experimental design, taking into account multiple relevant factors and providing more accurate feedback as to the specific responses perturbations in these factors invoke (Timmusk et al., 2017).

A significant hindrance in analysis of the drought root microbiome is the methodology used to elucidate the effect of soil moisture on the root-associated bacterial communities. Relatively few studies have examined root microbiome in the context of an experimental drought; instead, seasonality and associated changes in rainfall and soil water content are used as a proxy. Unfortunately, while in such studies soil moisture is indeed significantly different between seasons, presumably there are a number of environmental variables that similarly differ between seasons that represent confounding factors that cannot be separated from soil moisture, such as atmospheric [CO_2_] and soil temperature [both of which are confirmed to exert effects on soil bacterial communities (Hayden et al., 2012; Gunnigle et al., 2017)]. For this reason, future studies of drought’s effect on root bacterial communities should be conducted in the presence of an experimental drought in order to draw meaningful conclusions.

Plant response to drought involves a number of dynamic phenotypic modifications that will differ depending on how long they have been under water limitation. Thus, it might be expected that the length of exposure to drought will affect the root microbiome, such that community composition at drought onset could be drastically different from that seen weeks or months later. It has already been demonstrated that root and rhizosphere assemblages are affected by plant age and developmental stage (Chaparro et al., 2014; Wagner et al., 2016), indicating that even under control watering conditions there are shifts in the microbiome over time. With respect to drought, root samples taken from droughted cereal grasses pre- and post-flowering (where the latter group was not only under a different developmental stage but had been exposed to drought for an additional 5 weeks) indicated significant differences by time point (Naylor et al., 2017). A true time series for the root microbiome over the course of drought exposure and plant development would serve to highlight significant trends in accumulation or depletion of bacterial taxa, as well as the effect of plant developmental stage on microbiome recruitment under drought. Furthermore, as discussed, community abundance and diversity responses will vary based on drought history of the local environment (Bouskill et al., 2013, 2016a; Göransson et al., 2013), In future experiments, noting the environmental context and developmental stage a study is conducted in would be essential for explaining potential discrepancies with other research.

With respect to the bacterial community under drought, a question that warrants future investigation is: what exactly are the criteria that lead to proliferation of certain bacterial taxa in water-limited systems? In our review, we have extensively discussed divisions that might influence drought enrichment, including Gram-positive vs. Gram-negative, monoderm vs. diderm, oligotrophy vs. copiotrophy, and presence vs. absence of known PGP traits. However, none of these traits are universally linked with drought enrichment: for example, Proteobacteria are almost universally Gram-negative, yet members of Proteobacteria have been found in core droughted root microbiomes (Coleman-Derr et al., 2016; Naylor et al., 2017). Thus, broad conclusions about susceptibility or tolerance of a given group of bacteria should be made carefully, as the basis for drought enrichment may in some cases be more complex than the presence of a certain single trait.

In this review, we have attempted to summarize existing knowledge regarding the complex interplay between soils, plants, microbes and drought, which ultimately act to determine root-associated bacterial community composition. Plants are non-autonomous systems in their ecosystems, and much of their functioning, including nutrient uptake and stress response, is linked to soil bacterial communities. However, despite the importance of the root microbiome on plant health, as well as the increasing frequency of drought events due to climate change, very little research is available on the root microbiome under drought. This gap exists in part due to our incomplete understanding of relative contributions of and interactions between the various factors that produce the resultant bacterial communities. Future refinement and consolidation of methods by which the drought-root microbiome is studied will lead to a much richer understanding of these processes. Insight into plant enrichment of bacterial taxa under drought will identify taxa implicated in plant growth promotion, and in turn enhance development of microbial-based soil amendment strategies to alleviate drought stress for crops in arid regions, thus boosting food security against the increasing threat of climate change.

## Author Contributions

DN organized and wrote the manuscript. DC-D edited the manuscript.

## Conflict of Interest Statement

The authors declare that the research was conducted in the absence of any commercial or financial relationships that could be construed as a potential conflict of interest.
